# Simulated-patient evaluations during formative interviews predict exam performance better than self-reported empathy and the ability to recognize others’ emotions

**DOI:** 10.1186/s12909-026-09934-w

**Published:** 2026-07-17

**Authors:** Valerie Carrard, Céline Bourquin, Sylvie Félix, Francine Viret, Yusuke L. Takeuchi, Alexandre Berney

**Affiliations:** 1 Psychiatric Liaison Service, Department of Psychiatry, Lausanne University Hospital (CHUV) and University of Lausanne, Av. de Beaumont 23, Lausanne, 1011 Switzerland; 2 Present Address: School of Medicine, Faculty of Biology and Medicine, University of Lausanne, Rue Dr. César Roux 19, Lausanne, 1005 Switzerland

**Keywords:** Medical student, Communication skills, Empathy, Emotion recognition, Simulated patient, OSCE

## Abstract

**Background:**

Communication skills are central to clinical practice and are typically assessed through Objective Structured Clinical Examinations (OSCEs). Prior research indicates that self-reported questionnaires, simulated-patient evaluations, and expert assessments each offer valuable yet non-interchangeable perspectives on medical students’ communication skills. However, it remains unclear whether these assessment modalities, when collected during the academic year, can predict subsequent OSCE performances and thereby help identify students struggling to meet communication competency standards. This study examined whether self-reported empathy, the ability to recognize others’ emotions, and simulated-patient evaluations of communication skills predict later OSCE performances in medical students.

**Methods:**

Data were drawn from the ETMED‑L longitudinal open-cohort project at the University of Lausanne. The third- and fifth-year medical students who completed the yearly ETMED‑L online questionnaire and consented to the use of their communication skills evaluations were eligible for the present study. Self-reported empathy was assessed using validated multidimensional instruments and the ability to recognize others’ emotions was measured with a performance-based task. Simulated patients used a standardized form to evaluate communication skills during mandatory formative interviews. OSCE performances were rated by experts using structured communication grids and total exam points. Structural equation modelling was used to examine how self-reported empathy, the ability to recognize others’ emotions, and simulated-patient evaluations independently and jointly predict OSCE performances.

**Results:**

A sample of 468 third‑year and 399 fifth‑year students were analyzed. In both cohorts, self‑reported empathy, the ability to recognize others’ emotions, and simulated-patient evaluations independently predicted OSCE performances. In combined models, only simulated-patient evaluations remained significant predictors of OSCE performances, with a moderate effect size for the third-year cohort and a small effect size for the fifth-year cohort.

**Conclusions:**

Simulated-patient evaluations of student communication skills during formative training are more strongly associated with later expert-rated OSCE performances than both self-reported empathy and a performance-based measure of the ability to recognize others’ emotions. These findings underscore the practical value of structured simulated‑patient programs within competency‑based medical curricula, as they may help identify students who have difficulty attaining required communication competencies.

**Supplementary Information:**

The online version contains supplementary material available at https://doi.org/10.1186/s12909-026-09934-w.

## Introduction

Effective communication skills are an essential pillar of medical competence. Since 2018, Switzerland has adopted a national competency-based approach to communication learning outcomes [1], aligned with the CanMEDS competency framework [2]. Guided by societal and patient needs, it defines specific competencies that medical students should have developed by the end of their undergraduate education. These include communication skills that are described through key dimensions such as “empathy”, “active listening”, “mutual understanding”, and “a patient-centered approach to communication” [3]. In this framework, Objective Structured Clinical Examinations (OSCEs) are used to evaluate medical students’ communication skills. OSCEs are standardized, performance-based examinations in which students participate in a sequence of structured clinical scenarios. In these stations, patients are portrayed by trained simulated patients, and students’ performance is assessed using standardized checklists completed by experts. Previous studies suggest that OSCE performances predict aspects of future clinical performance, including communication skills in clinical practice [4, 5].

Substantial research has examined how self-reported questionnaires, simulated-patient evaluations, and expert assessments of communication skills relate to one another, with the aim of testing the reliability across these assessment modalities and the possibility of integrating different evaluation sources in the OSCEs [6–14]. For self-reported questionnaires, past research has mostly focused on self-reported empathy because it has been defined as a central element to patient-centered communication and effective patient-provider communication [15, 16]. Past research provides mixed results regarding the relationship between self-reported empathy and OSCE performances. A meta-analysis reports that some studies found no significant link, whereas other found significant relationships for certain dimensions of empathy but not for others [17]. The ability to recognize others’ emotions is, by definition, a skill that facilitates empathy [18] and has the advantage of being readily measurable through performance-based tasks that do not rely on self-report. As such, it represents a promising avenue for more objective assessments of communication skills and preliminary evidence suggests that the ability to recognize others’ emotions assessed with performance-based tests is associated with better performance in OSCEs [3]. Regarding the relationship between simulated-patient evaluations, many cross-sectional studies have explored correlations between simulated-patient and expert assessments during the same OSCEs [6–12]. While findings generally show good overall agreement, they also consistently reveal discrepancies (see for instance [6–8]) as they capture distinct perspectives and sensitivities regarding different dimensions of communication skills.

Building on this conclusion of both agreement and complementarity, a logical extension of this work is to move from a cross-sectional to a longitudinal perspective by examining whether self-reported measures and/or simulated-patient ratings collected throughout an academic year can predict subsequent performance during the OSCEs. Addressing this question seems essential, as identifying predictive markers would have important practical implications for the early detection of students at risk of not reaching the expected level of communication skills. In turn, educators could explore these students’ pedagogical needs to foster improvement, thereby helping ensure that future physicians achieve the required level of competence in communication for high-quality clinical practice.

In the context of the ETMED-L project [19], a cohort study examining the evolution of mental health and empathy throughout medical school via yearly online questionnaires, we additionally collected simulated-patient evaluations during formative interviews as well as OSCE performances data. The aim of the present study was then to examine whether medical students’ self-reported empathy, their ability to recognize others’ emotions, and the simulated-patient evaluations of their communication skills during the academic year could predict subsequent OSCE performances as evaluated by experts.

## Methods

### Design

This study is part of the four-year ETMED-L project that examines the mental health and empathy of medical students using a longitudinal open-cohort design.

### Procedure

Each academic year from 2020-2021 to 2023-2024, an online questionnaire was sent to all medical students registered at the University of Lausanne (Switzerland), except for the external exchange students. The questionnaires were sent at the beginning of March 2021, November 2021, November 2022, and November 2023 (exam-free months). Each time, students had one month to complete the questionnaire, which took approximately one hour to complete. They received 50 CHF (~ 50 USD) upon completion of each yearly questionnaire. The online questionnaire [20] included several validated self-reported instruments, a performance-based test of the ability to recognize others’ emotions (see "Measures" section), and a consent form for the retrieval and use for research of the communication skills evaluations produced during their curriculum. If they gave their written consent for it, two types of communication skills evaluations were retrieved: simulated-patient evaluations and OSCE performances.

Within our 6-year medical curriculum, mandatory formative encounters with simulated patients begin in the second year with group sessions and continue annually with individual sessions until the fifth year. The simulated-patient evaluations of students’ communication skills used in the present study were conducted in curriculum year 3 (two formative interviews between September and May on the topic of history taking and diagnosis) and curriculum year 5 (one motivational interview between November and December).

OSCEs’ final grades and communication skills ratings were also retrieved for the present study. These OSCEs are mandatory qualifying exams of students’ clinical competences conducted in May of curriculum year 3 and March of curriculum year 5. During the two days of an OSCE, students rotate through eight different stations in which specific clinical situations are simulated (e.g., headache, cough, back, hip, chest, or abdominal pain). The clinical situations chosen are meant to represent a variety of contexts such as ambulatory and in-patient, acute and emergency medicine, as well as general symptoms, with one of the stations consistently presenting a scenario in which communication skills are particularly critical (e.g., managing anxiety or breaking bad news). Students’ performance in each clinical situation is assessed in a structured manner by experts using four evaluation grids: one for the quality of the history taking, one for the physical examination, one for the appropriateness of the proposed management, and one for communication skills. In the present study, we analyzed data from the communication skills evaluation grid and the final total OSCE points. Note that the same communication skills grid was used across all stations, and its score accounts for 25% of the final OSCE score.

The whole ETMED-L project, including the use of simulated-patient and expert evaluations, was reviewed and approved by the Cantonal Research Ethics Committee – Vaud (Commission cantonale d’éthique de la recherche sur l’être humain – Vaud [CER-VD]) an independent public ethics committee operating at the cantonal level in Switzerland (project number 2020–02474 approved on January 20, 2021). The study was conducted in accordance with the Swiss legal requirements [21, 22], the current version of the World Medical Association Declaration of Helsinki [22], and the principles and procedures for integrity in scientific research involving human beings. The privacy rights of human subjects have been observed, and all participants provided written informed consent.

### Participants

For the present study, two cohorts were extracted from the ETMED-L data: the Year 3 and the Year 5 cohorts. They included all medical students who filled in the ETMED-L questionnaire during their third or fifth curriculum year and gave written consent for the retrieval and use of their communication skills evaluations. Note that the participants of the first ETMED-L data collection wave (2020–2021 academic year) were excluded, because simulated-patient evaluations were only implemented from the second data collection year on.

### Measures

#### Online questionnaire data

The ETMED-L questionnaire [20] included a section related to sociodemographic information, self-reported instruments assessing different dimensions of empathy, and a performance task of the ability to recognize others’ emotions. It also presented several measures of mental health and burnout that were not used in the present study.

##### Control variables

Seven sociodemographic characteristics known to potentially influence empathy and communication skills were used as control variables in the present study: gender identification (male, female, or non-binary), age, number of parents with higher education (college or university degree), social support (mean score of two items measuring self-perceived availability of emotional and practical support from 0="not at all” to 10="a great deal”), having a partner, number of hours of physical activity per week, and having consulted a psychotherapist in the past year.

##### Self-reported empathy

The French versions of three well-validated instruments measuring different dimensions of empathy were used. The widely used Jefferson Scale of Physician Empathy Student-Version (JSPE-S [23]) was developed to assess medical students’ orientations or attitudes toward empathic relationships in the context of patient care and thus measures the cognitive dimension of empathy (Cronbach’s α = 0.78). The Questionnaire of Cognitive and Affective Empathy (QCAE [24, 25]) was validated in a large sample of university students, and both the English and the French versions have been shown to reliably assess the cognitive and affective dimensions of empathy separately (Cronbach’s α = 0.86 and 0.81, respectively for cognitive and affective dimensions). The Ability to Modify Self-Presentation Scale (AMSP [26]) assesses one’s ability to adapt expressive behaviors in different social situations and was thus chosen as a self-reported measure of the behavioral dimension of empathy (Cronbach’s α = 0.81).

##### Ability to recognize others’ emotions

The Geneva Emotion Recognition Test short version (GERT-S [27]) is a performance task presenting 42 short videos (less than 30 s each) of actors portraying one out of 14 different emotions (e.g., fear, despair, surprise, disgust, anger). The score is then computed as the percentage of emotions correctly recognized. A meta-analysis concluded that the GERT shows among the highest reliability estimates and strongest convergence with other emotion recognition measures among interpersonal accuracy tests [28].

#### Simulated-patient evaluations

All medical students were informed by email that, as part of the ETMED-L project, simulated patients would evaluate their communication skills after each formative interview on a paper-pencil form developed for this study (see Supplementary Document 1). The form included the 5-item Jefferson Scale of Patient Perception of Physician Empathy (JSPPPE [29]), a single item regarding general satisfaction with the communication (from 1 = unsatisfactory to 5 = exceptionally satisfactory), and the communication skills grid used during the OSCEs that includes 4 items relating to the quality of (1) response to the patient’s feelings and needs, (2) structure of the interview, (3) verbal expression, and (4) non-verbal expression (rated from 1 = not at all to 5 = totally).

#### OSCE performances

The total OSCE points on which the final exam grade is based has been retrieved for the present study. Moreover, the communication skills grid filled in by all experts in every station was averaged across stations. It included a single item regarding general communication competency (from 1 = incompetent to 5 = exceptionally competent) and the same 4 items as in the simulated patients’ communication form: (1) response to the patient’s feelings and needs, (2) structure of the interview, (3) verbal expression, and (4) non-verbal expression (see Supplementary Document 2 for the detailed OSCE communication skills evaluation form). The distribution, as well as the skewness and kurtosis indices for each OSCE performance indicator, are provided in the supplementary material (Supplementary Fig. 1 and Supplementary Table 1). They revealed no significant deviation from normality and no ceiling effect.

### Statistical analyses

**Fig. 1:**
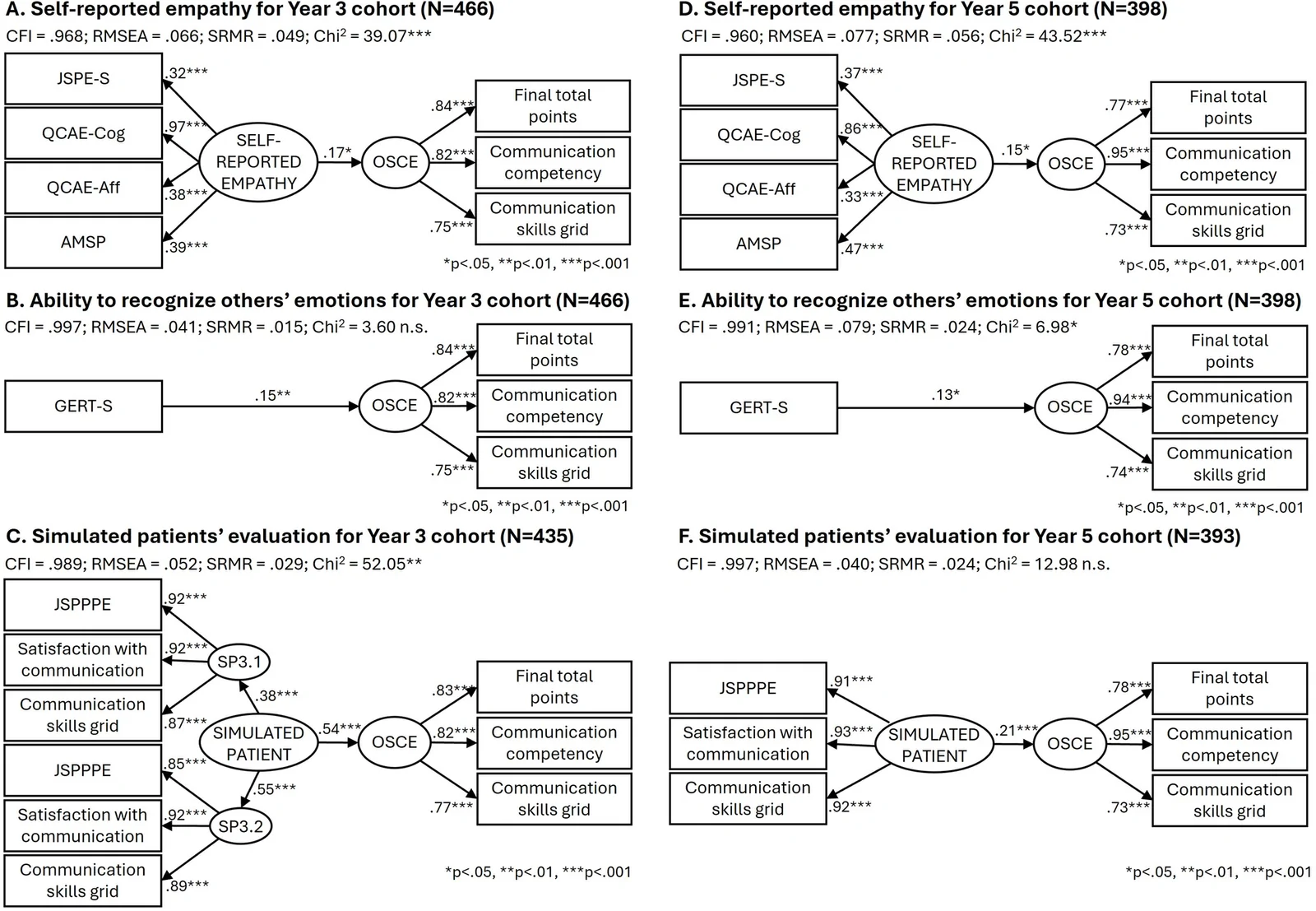
Structural equation models with self-reported empathy, ability to recognize others’ emotions, and simulated-patient evaluations independently predicting exam performance. Panel **A to C** present Year 3 students (*N* = 432), and Panel **D to F** present Year 5 students (*N* = 392). Circles represent latent variables and rectangles represent observed variables. Arrows indicate standardized β regression coefficients (thresholds of 0.1, 0.3, and 0.5 interpreted respectively as small, medium, and large effect sizes), with asterisks denoting statistical significance (*p < .05, **p < .01, ***p < .001). All models showed acceptable fit to the data as indicated by the CFI (Comparative Fit Index) > 0.90, RMSEA (Root Mean Square Error of Approximation) < 0.05, and SRMR (Standardized Root Mean Square Residual) < 0.08. The Chi2 test is reported but not used as a decisional index. Abbreviations: SP = simulated-patient evaluations; OSCE = Objective Structured Clinical Examination; JSPE-S = Jefferson Scale of Physician Empathy - Student version; QCAE = Questionnaire of Cognitive and Affective Empathy; AMSP = Ability to Modify Self-Presentation; GERT-S = Geneva Emotion Recognition Test - Short Version; JSPPPE = Jefferson Scale of Patient Perception of Physician Empathy

**Fig. 2:**
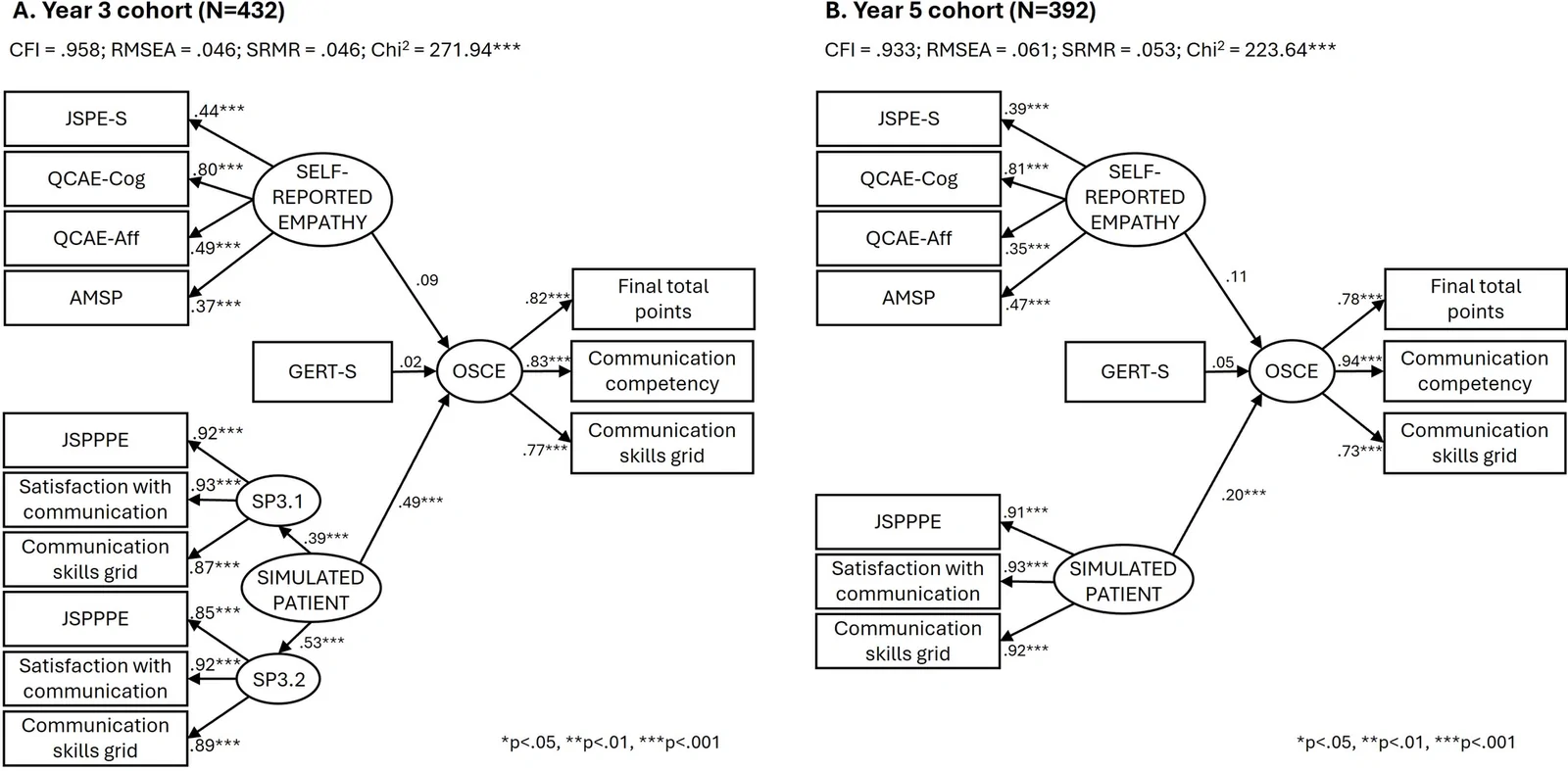
Structural equation models with self-reported empathy, ability to recognize others’ emotions, and simulated-patient evaluations independently predicting exam performance. Panel A presents Year 3 students (N = 432), and Panel B presents Year 5 students (N = 392). Circles represent latent variables and rectangles represent observed variables. Arrows indicate standardized β regression coefficients (thresholds of 0.1, 0.3, and 0.5 interpreted respectively as small, medium, and large effect sizes), with asterisks denoting statistical significance (*p < .05, **p < .01, ***p < .001). Control variables (identifying as male, age, parents’ education, social support, having a partner, physical activity, and having consulted a psychotherapist) were included but are not shown for clarity. All models showed acceptable fit to the data as indicated by the CFI (Comparative Fit Index) > 0.90, RMSEA (Root Mean Square Error of Approximation) < 0.05, and SRMR (Standardized Root Mean Square Residual) < 0.08. The Chi2 test is reported but not used as a decisional index. Abbreviations: SP = simulated-patient evaluations; OSCE = Objective Structured Clinical Examination; JSPE-S = Jefferson Scale of Physician Empathy - Student version; QCAE = Questionnaire of Cognitive and Affective Empathy; AMSP = Ability to Modify Self-Presentation; GERT-S = Geneva Emotion Recognition Test - Short Version; JSPPPE = Jefferson Scale of Patient Perception of Physician Empathy

Most assessment types examined in this study (self-reported empathy, simulated-patient evaluation, and OSCEs) were evaluated using multiple indicators. Consequently, structural equation modeling (SEM) with latent variables was employed (see also Figs. 1 and 2 for graphical representation of the models). This approach allows for the effective and efficient modeling of complex interrelationships among multiple variables. The SELF-REPORTED EMPATHY latent variable was estimated using scores from the JSPE-S, the cognitive and affective dimensions of the QCAE, and the AMSP. The SIMULATED PATIENT latent variable was estimated using the JSPPPE scores, the satisfaction with communication item, and the communication skills grid scores. For the Year 3 cohort, simulated-patient evaluations occurred during two formative interviews; thus, these indicators first loaded onto two intermediate latent variables (SP3.1 and SP3.2), which then loaded onto the SIMULATED PATIENT latent variable. The latent outcome variable, OSCE, was estimated using the final total OSCE points, the communication competency item, and the communication skills grid scores.

First, to determine whether self-reported empathy, the ability to recognize others’ emotions, and simulated-patient evaluations of medical students’ communication skills predict expert-rated OSCE performances, six separate models were tested: 2 cohorts (Year 3 and Year 5) X 3 assessment types (SELF-REPORTED EMPATHY, GERT-S [ability to recognize others’ emotions], and SIMULATED PATIENT) independently predicting exam performance (OSCE). Then, to test the conjoint impact of all assessment types, one final model was computed for each cohort separately (Year 3 and Year 5) with SELF-REPORTED EMPATHY, GERT-S (ability to recognize others’ emotions), and SIMULATED PATIENT evaluations predicting conjointly OSCE. Moreover, identifying as male, age, parents’ education, social support, having a partner, physical activity, and having consulted a psychotherapist were included in the models as control variables predicting SELF-REPORTED EMPATHY, GERT-S, SIMULATED PATIENT, and OSCE.

In all models, Maximum Likelihood estimation with Robust standard errors (MLR) was used. Goodness of fit of the models was evaluated with the following indices and rules: the Comparative Fit Index (CFI) should be > 0.90, the Root Mean Square Error of Approximation (RMSEA) should be < 0.05, and the Standardized Root Mean Square Residual (SRMR) should be < 0.08 [30]. The classic Chi^2^ test was reported, but not used as a decisional index, because it is notably sensitive to sample size and thus nearly always rejects the model when large samples are used [31]. Effect sizes were estimated using the standardized regression coefficient β, with thresholds of 0.1, 0.3, and 0.5 interpreted as small, medium, and large effect sizes, respectively [32].

The highest missing rate in our data was 4.27% (simulated patients’ satisfaction with communication item in Year 3 cohort). Since research indicates that minimal information gain is achieved from imputing missing data when the missing rate is below 5% [33], listwise deletion was applied in all analyses. All analyses were run in Stata 18 [34].

## Results

### Sample

A detailed flow chart is available as supplementary material (Supplementary Fig. 2). Among the 756 eligible third-year students, 528 participated to the ETMED-L project and 488 gave their consent for the retrieval and use of their communication skills data. Then, nine students who gave a wrong answer to the attention check questions of the questionnaire were excluded. After excluding students for whom communication skills data could not be retrieved (*n* = 10) or with totally missing self-reported empathy and ability to recognize others’ emotions scores (*n* = 1), the final Year 3 cohort sample included 468 students, representing 62% of the eligible students. For the Year 5 cohort, 718 fifth-year students were eligible, 526 participated to the ETMED-L project and 474 gave their consent for the retrieval and use of their communication skills data. After exclusion of the students who failed the attention check questions (*n* = 7) and those for whom communication skills data could not be retrieved (*n* = 68), the final Year 5 cohort sample included 399 students, representing 56% of the eligible students.

### Descriptive statistics

The sample descriptives are presented in Table 1. The participants are well distributed across the three participation waves, with between 30% and 36% of the students in each ETMED-L data collection year. The included students were 23 years old on average (range: 19–51) and represent well the gender proportions usually observed in the Lausanne Medical School, with a majority identifying as female (69%), a smaller proportion identifying as male (32%), and a minority identifying as non-binary (1%). Regarding sociodemographic background, around half of the included students have two parents with higher education (56%), and half of the students do not have a paid job in addition to their studies (54%). Detailed statistics for the variables of interest are available in Supplementary Tables 1 and correlations between the predicting variables are displayed in Supplementary Table 2.

**Table 1:** Sample Descriptives

	Year 3 (*N* = 468)		Year 5 (*N* = 399)	
	*N*	%	*N*	%
Participation year				
* 2021–2022*	151	32.26	106	26.57
* 2022–2023*	169	36.11	143	35.84
* 2023–2024*	148	31.62	150	37.59
Gender identification				
* Female*	321	68.59	259	64.91
* Male*	144	30.77	135	33.83
* Non-binary*	3	0.64	5	1.25
Parents with higher education				
* Both*	260	55.56	224	56.14
* One*	109	23.29	82	20.55
* None*	99	21.15	93	23.31
Having a partner				
* Yes*	246	52.56	226	56.64
* No*	222	47.44	173	43.36
Having consulted a psychotherapist				
* Yes*	100	21.37	109	27.32
* No*	368	78.63	290	27.32

### Structural equation models’ results

Results of the structural equations models testing how self-reported empathy, ability to recognize others’ emotions, and simulated-patient evaluations independently predict OSCE performances are displayed in Fig. 1 (see also Supplementary Tables 3 and 4 for detailed results). We observed that in both cohorts (Year 3 and 5) all three assessment types significantly predicted expert-rated OSCE performances. For the Year 3 cohort, simulated-patient evaluations demonstrated a strong significant effect (β = 0.54, *p*<.001), while self-reported empathy (β = 0.17, *p*=.013) and the ability to recognize others’ emotions (β = 0.15, *p*=.002) showed smaller but significant effects. In the Year 5 cohort, all predictors yielded small significant effect sizes (self-reported empathy: β = 0.15, *p*=.039; ability to recognize others’ emotions: β = 0.13, *p*=.013; simulated-patient evaluations: β = 0.21, *p*<.001). All models showed a good fit to the data on all indices considered.

Figure 2 presents the results of the structural equation models examining how self-reported empathy, ability to recognize others’ emotions, and simulated-patient evaluations conjointly predict OSCE performances (see Supplementary Tables 5 and 6 for detailed results). These analyses indicate that simulated-patient evaluations are more strongly associated with OSCE performance than the other variables examined. When considered alongside simulated-patient evaluations, neither self-reported empathy nor the ability to recognize others’ emotions significantly predict OSCE performances. In contrast, simulated-patient evaluations show a significant association, with an effect size approaching large for the Year 3 cohort (β = 0.49, *p*<.001) and small for the Year 2 cohort (β = 0.20, *p*<.001). All indices indicated a good fit to the data.

## Discussion

The present study examined whether self-reported empathy, the ability to recognize others’ emotions, and simulated-patient evaluations during the academic year predict future exam performance. Our findings indicate that, among these three assessment modalities, simulated-patient evaluations obtained during formative training are the most strongly associated with future exam results.

Indeed, when self-reported empathy and the ability to recognize others’ emotions were included in the same model as simulated-patient evaluations, only simulated-patient evaluations remained a significant predictor of future OSCE performances. This result may stem not only from the conceptual alignment between their rating schemes and those used in the exams, but also from the high ecological validity of interpersonal interactions. Communication skills are inherently relational and context-dependent; thus, assessments embedded in real-time interactions capture nuances that static measures like questionnaires and performance tasks cannot. The results of the present study thus align with past cross-sectional literature underscoring the value of simulated-patient evaluations as a reliable measure of students’ communication skills that is consistent with experts’ ratings [6–12]. Importantly, our study extends this literature by providing longitudinal evidence that simulated-patient evaluations collected during the academic year predict subsequent OSCE performances.

This convergence between simulated patients and experts may be attributed to the comprehensive training that simulated patients receive at our institution. At the University of Lausanne, the simulated-patient training program includes theoretical introductions, role-plays, feedback giving skills, debriefing, and regular evaluations [35], all of which help ensure alignment with the communication competencies expected from experts during exams. The specificity of the simulated-patient evaluation additionally resides in the combination of a trained and structured perspective with a subjective patient experience that unfolds in the interaction with the student. The importance of simulated patients’ feedback – and of their training to provide it – is well established [36]. Consistent with experiential learning theory [37], which posits that interpersonal competencies are best developed through authentic practice, structured feedback, and opportunities for reflection. Research has shown that simulation-based medical education is most effective when it incorporates accurate and meaningful feedback [36]. Further studies comparing trained and untrained simulated patients are needed to clearly estimate the impact of training quality on the congruence between simulated-patient and expert evaluations. Nevertheless, the results of the present study could suggest that, beyond the pedagogical value of accurate feedback in itself, appropriate training could additionally enable simulated patients to provide evaluations that are congruent with expert assessments during OSCEs when using a comparable scoring grid.

Indeed, the other important practical implication stemming from the predictive power of simulated-patient evaluations is that these assessments could help proactively identify students demonstrating early signs of difficulty in achieving required communication skills standards. An important next step would therefore be to adopt a categorical approach, by defining a validated threshold for OSCE communication performance and using methods such as logistic regression to evaluate predictive utility of simulated-patient evaluations in distinguishing lower-performing learners. If its predictive potential is confirmed, this early identification would enable targeted pedagogical interventions. However, translating these insights into action remains complex, as the underlying causes of underperformance can vary substantially from one student to another. Consequently, any interventions would need to be carefully designed and tailored to address each learner’s specific needs. They would also need to be embedded in a process of reflective integration by the student in order to be effective [38] and, ultimately, to support the development of the nuanced communication skills required for quality clinical practice.

Although simulated-patient evaluation is more strongly associated with exam performance, our results also show that self-reported empathy is a significant, though modest, predictor of exam performance when considered on its own. Past findings regarding the link between self-reported empathy and expert-rated performance are mixed, with some studies showing moderate but significant associations [39, 40] and others finding no significant link [7]. The significant link found in the present study may stem from the multidimensional approach adopted to measure empathy. Empathy is widely recognized as a multidimensional construct, encompassing at least cognitive (i.e., perspective-taking) and affective processes (i.e., emotional contagion). By incorporating multiple instruments (JSPE-S, QCAE, and AMSP) assessing three dimensions of empathy (cognitive, affective, and behavioral), our study may have provided a more comprehensive self-reported measure of empathic ability that better aligns with expert ratings during OSCEs.

The ability to recognize others’ emotions, measured through a performance-based task that does not rely on self-report was also a significant predictor of OSCE performances when considered on its own. Aligning with the very scarce existing literature [3], our findings show that the ability to recognize others’ emotions is indeed significantly associated with exam performance, with a small effect size. This result is encouraging, as it suggests that the capacity to accurately identify others’ emotions translates into observable behaviors during OSCEs that experts seem able to capture in their evaluations. It thus provides some support for the accuracy of expert assessments during OSCEs.

It is also worth noting that simulated-patient evaluations and OSCE performances likely reflect a partially overlapping construct, as they rely on similar rating scales. In contrast, self-reported empathy and the ability to recognize others’ emotions represent more distinct constructs. Therefore, even if smaller in magnitude, the contribution of self-reported empathy and the ability to recognize others’ emotions to OSCE performances should not be underestimated.

### Limitations and strengths

The present study’s strength lies in the variety of communication skills evaluations included, the multidimensional approach to empathy, the use of validated instruments, and the collection of assessments grounded in practical teaching (simulated-patient interviews and OSCEs). However, the design of this study comes with some limitations that should be acknowledged. Although the response rate achieved was at the higher end of what is typically observed in this population [41], participation bias may have compromised representativeness and, consequently, the generalizability of the findings. To address this, we compared our sample to the usual gender and curriculum year distribution observed at our university and included control variables in the final model to mitigate potential bias. Nevertheless, the results may not be fully applicable to populations known to have lower survey response rates. This study was conducted at a single institution, which may limit the generalizability of the findings to other medical schools or cultural contexts. Comparative studies across institutions are warranted to confirm the results and would also clarify the role of simulated-patient training protocols in shaping their evaluation accuracy.

### Conclusion

This study demonstrates that among different assessments of communication skills collected throughout the medical curriculum, simulated-patient evaluations showed the strongest and most consistent association with subsequent expert-rated OSCE performances. While self-reported empathy and the ability to recognize others’ emotions show modest associations with exam outcomes, only simulated-patient evaluations retain predictive value when considered alongside other measures, highlighting their ecological validity and their capacity to capture relational, context-dependent aspects of communication. These findings support the integration of structured simulated-patient programs not only as formative training tools but also as valuable contributors to competency-based assessment strategies, enabling earlier identification of students who may require pedagogical support. By reinforcing the role of experiential and multisource assessments, this study underscores the importance of maintaining high-quality simulated-patient training and rating to foster the development of communication skills essential for future clinical practice.

## Supplementary Information

Supplementary Material 1.

## Data Availability

The dataset supporting the conclusions of this article is available in the Zenodo repository, https://doi.org/10.5281/zenodo.19250423.
