# Supplementary material for: Simulated-patient evaluations during formative interviews predict exam performance better than self-reported empathy and the ability to recognize others’ emotions
Source: BMC Med Educ. 2026 Jul 17;26:1630. doi: 10.1186/s12909-026-09934-w (PMC13660341; doi:10.1186/s12909-026-09934-w)
Supplement: Supplementary file 1 [file 12909_2026_9934_MOESM1_ESM.pdf]

Supplementary material for the paper:

Simulated-patient evaluations during formative interviews predict exam performance better than self-reported empathy and the ability to recognize others' emotions

Valerie Carrard, Céline Bourquin, Sylvie Félix, Francine Viret, Yusuke L. Takeuchi, Alexandre Berney

Supplementary Document 1. Simulated-patient evaluation form

Supplementary Document 2. OSCE communication skills evaluation form

Supplementary Figure 1. Distribution of OSCE performance measures

Supplementary Figure 2. Flow Chart

Supplementary Table 1. Detailed statistics for variables of interest

Supplementary Table 2. Correlations between self-reported empathy, the ability to recognize others' emotions, and simulated-patient evaluation

Supplementary Table 3. Detailed results of Year 3 cohort's structural equation models with self-reported empathy, ability to recognize others' emotions, and simulated patient evaluations predicting independently exam performance.

Supplementary Table 4. Detailed results of Year 5 cohort's structural equation models with self-reported empathy, ability to recognize others' emotions, and simulated patient evaluations predicting independently exam performance.

Supplementary Table 5. Detailed results of Year 3 cohort's structural equation models with self-reported empathy, ability to recognize others' emotions, and simulated patient evaluations predicting conjointly exam performance (N=432).

Supplementary Table 6. Detailed results of Year 5 cohort's structural equation models with self-reported empathy, ability to recognize others' emotions, and simulated patient evaluations predicting conjointly exam performance (N=392).

Supplementary Document 1. Simulated-patient evaluation form

	Not at all 1	2	3	4	Totally 5
**1. Response to the patient's feelings and needs**<br>Responds insightfully and appropriately to the patient's cues and needs (verbal or non-verbal)	☐	☐	☐	☐	☐
**2. Structure of the interview**<br>Clear interaction plan throughout the consultation, the plan is coherent and flexible, the student conducts the interview consistently	☐	☐	☐	☐	☐
**3. Verbal expression**<br>Communicates in an appropriate way that promotes understanding, adapts communication to the patient (vocabulary suited to sociocultural level, repetition, summaries, articulation, tone, etc.)	☐	☐	☐	☐	☐
**4. Non-verbal expression**<br>Enhances the relationship with the patient and their participation through eye contact, gestures, posture, interpersonal distance, pauses, etc.	☐	☐	☐	☐	☐

**During the interview, I had the impression that this student:**	Totally disagree 1	2	3	4	Totally agree 5
Items of the Jefferson Scale of Patient Perception of Physician Empathy (JSPPPE) are not displayed for copyright reasons.<br>*© Thomas Jefferson University. All rights reserved.*	☐	☐	☐	☐	☐

**General evaluation of communication from the SP's point of view**				
☐<br>**1**<br>**Unsatisfactory**	☐<br>**2**<br>**Borderline**	☐<br>**3**<br>**Satisfactory**	☐<br>**4**<br>**Very satisfactory**	☐<br>**5**<br>**Exceptionally satisfactory**

Supplementary Document 2. OSCE communication skills evaluation form

A. Communication skills grid

**1.**	**Response to the patient's feelings and needs**<br>Responds insightfully and appropriately to the patient's cues and needs (verbal or non-verbal)	
	5	totally
	4	
	3	
	2	
	1	not at all
**2.**	**Structure of the interview**<br>Clear interaction plan throughout the consultation, the plan is coherent and flexible, the candidate conducts the interview consistently	
	5	totally
	4	
	3	
	2	
	1	not at all
**3.**	**Verbal expression**<br>Communicates in an appropriate way that promotes understanding, adapts communication to the patient (vocabulary suited to sociocultural level, repetition, summaries, articulation, tone, etc.)	
	5	totally
	4	
	3	
	2	
	1	not at all
**4.**	**Non-verbal expression**<br>Enhances the relationship with the patient and their participation through eye contact, gestures, posture, interpersonal distance, pauses, etc.	
	5	totally
	4	
	3	
	2	
	1	not at all

B. Communication competency

	**General evaluation of communication**
5	exceptionally competent
4	very competent
3	competent
2	borderline
1	incompetent

Supplementary Figure 1. Distribution of OSCE performance measures

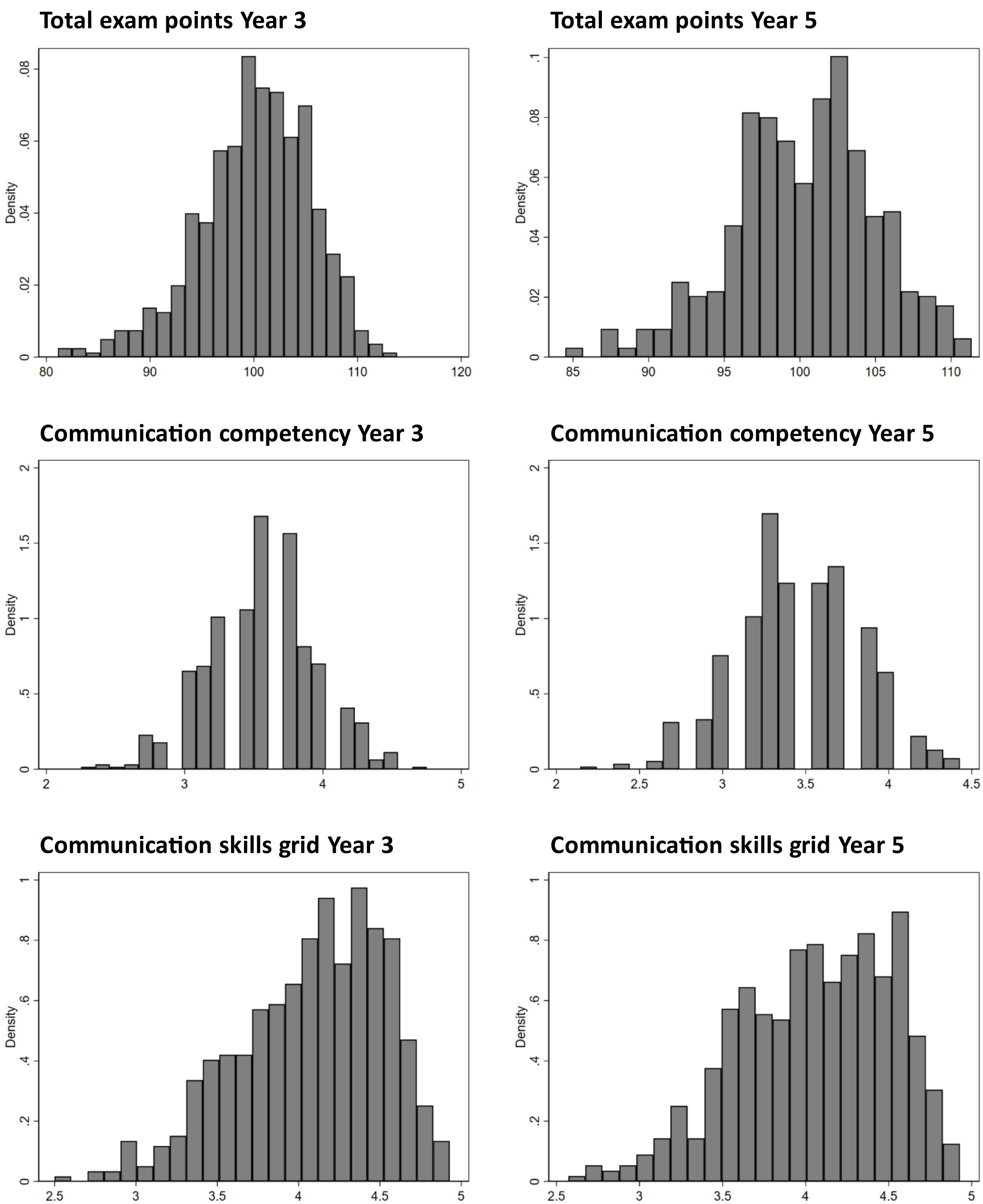

Supplementary Figure 2. Flow Chart

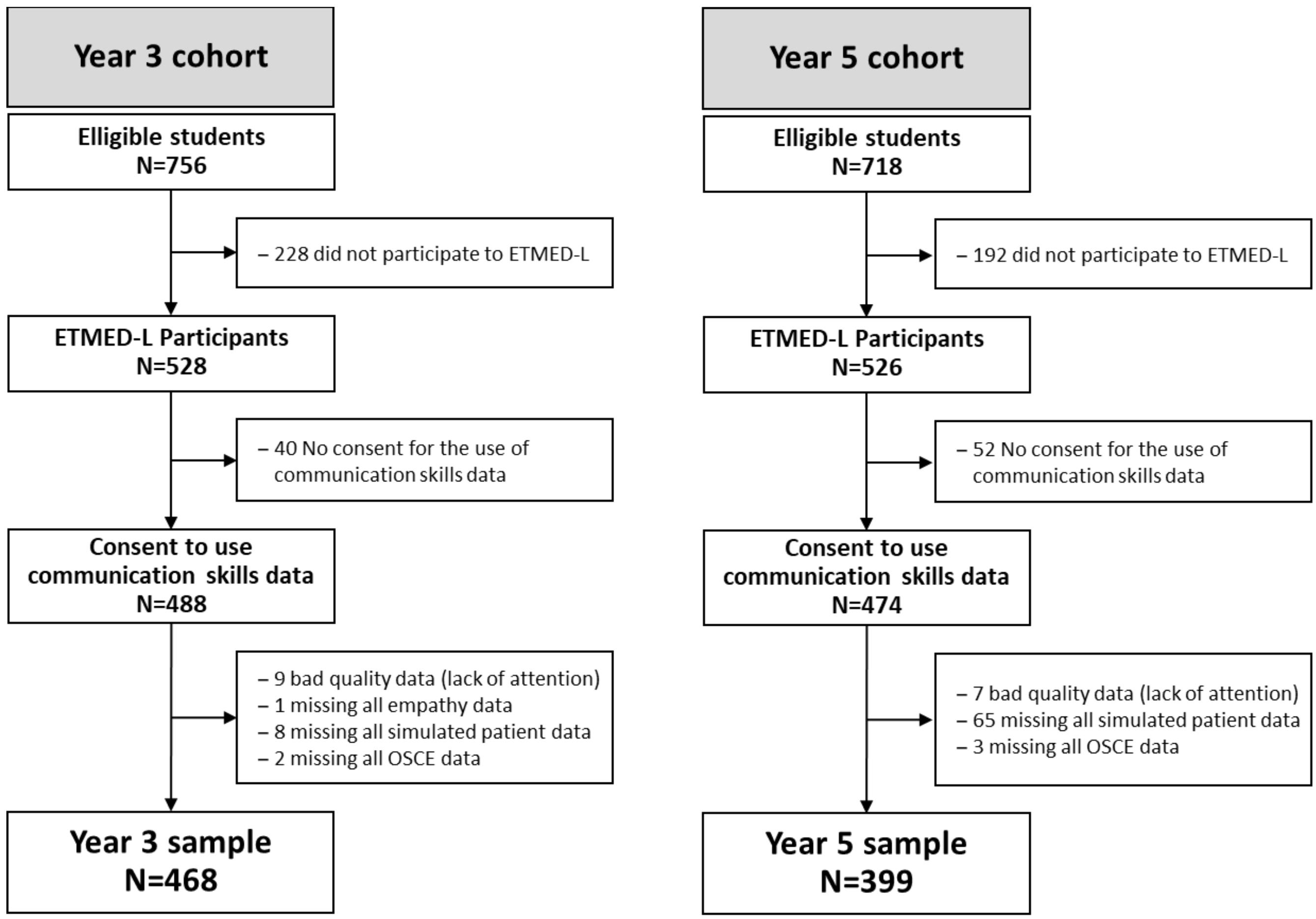

Supplementary Table 1. Detailed statistics for variables of interest

	Year 3					Year 5				
	N	Mean	SD	Skewness	Kurtosis	N	Mean	SD	Skewness	Kurtosis
**Self-reported empathy**										
JSPE-S	466	107.40	8.76	-0.64	3.65	398	108.64	7.94	-0.48	3.27
QCAE - Cognitive	468	58.33	6.92	-0.09	3.37	399	58.66	6.57	0.14	3.10
QCAE - Affective	468	34.21	5.76	-0.22	3.07	399	34.79	5.39	-0.14	3.18
AMSP	466	22.94	5.32	-0.19	3.25	398	22.95	4.86	0.04	2.89
**Ability to recognize others' emotions**										
GERT-S	466	0.72	0.11	-0.54	3.37	398	0.76	0.10	-0.89	5.51
**SP3.1**										
JSPPPE	453	3.71	0.77	-0.28	2.78	.	.	.	.	.
Satisfaction with communication	448	3.72	0.71	-0.22	2.78	.	.	.	.	.
Communication skills grid	453	3.86	0.69	-0.34	2.79	.	.	.	.	.
**SP3.2**										
JSPPPE	460	3.42	0.82	-0.17	2.75	.	.	.	.	.
Satisfaction with communication	455	3.45	0.79	-0.24	2.98	.	.	.	.	.
Communication skills grid	460	3.59	0.79	-0.33	2.96	.	.	.	.	.
**SP**										
JSPPPE	.	.	.	.	.	398	3.49	0.83	-0.10	2.51
Satisfaction with communication	.	.	.	.	.	394	3.52	0.83	-0.21	3.02
Communication skills grid	.	.	.	.	.	399	3.72	0.79	-0.34	2.82
**OSCE**										
Total exam points	468	100.17	5.33	-0.55	3.25	399	100.61	4.69	-0.30	3.13
Communication competency	468	3.54	0.37	-0.12	2.81	399	3.49	0.36	-0.11	2.97
Communication skills grid	468	4.13	0.44	-0.67	3.02	399	4.15	0.41	-0.55	3.03

Abbreviations: SP = simulated patients' evaluations; OSCE = Objective Structured Clinical Examination; JSPE-S = Jefferson Scale of Physician Empathy - Student version; QCAE = Questionnaire of Cognitive and Affective Empathy; AMSP = Ability to Modify Self-Presentation; GERT-S = Geneva Emotion Recognition Test - Short Version; JSPPPE = Jefferson Scale of Patient Perception of Physician Empathy.

Supplementary Table 2. Correlations between self-reported empathy, the ability to recognize others' emotions, and simulated-patient evaluation

**Year 3**	**SP3.1**			**SP3.2**		
	JSPPPE	***Satisfaction with communication***	***Communication skills grid***	***JSPPPE***	***Satisfaction with communication***	***Communication skills grid***
**Self-reported empathy**						
JSPE-S	.05	.00	.03	.03	.08	.06
QCAE - Cognitive	.08	.08	.08	.08	.09	.07
QCAE - Affective	.13	.13	.17	.14	.10	.09
AMSP	.06	.02	.04	.04	.06	.03
**Ability to recognize others' emotions**						
GERT-S	.03	.04	.07	.07	-.02	.03

**Year 5**	**SP**		
	JSPPPE	***Satisfaction with communication***	***Communication skills grid***
**Self-reported empathy**			
JSPE-S	.08	.06	.06
QCAE - Cognitive	.00	.00	-.04
QCAE - Affective	-.01	-.01	.01
AMSP	-.03	-.07	-.05
**Ability to recognize others' emotions**			
GERT-S	.09	.05	.11

Abbreviations: SP = simulated patient evaluations; JSPE-S = Jefferson Scale of Physician Empathy - Student version; QCAE = Questionnaire of Cognitive and Affective Empathy; AMSP = Ability to Modify Self-Presentation; GERT-S = Geneva Emotion Recognition Test - Short Version; JSPPPE = Jefferson Scale of Patient Perception of Physician Empathy.

Supplementary Table 3. Detailed results of Year 3 cohort's structural equation models with self-reported empathy, ability to recognize others' emotions, and simulated patient evaluations predicting independently exam performance.

	Std. Coef.	SE	z	p	95% CI
**MODEL 1 – SELF-REPORTED EMPATHY (N=466)**					
**Structural**					
OSCE ←					
SELF-REPORTED EMPATHY	0.17	0.07	2.48	.013	0.03 - 0.30
**Measurement**					
OSCE →					
Final total points	0.84	0.02	37.09	<.001	0.80 - 0.89
Communication competency	0.82	0.02	39.01	<.001	0.78 - 0.86
Communication skills grid	0.75	0.03	28.75	<.001	0.70 - 0.80
SELF-REPORTED EMPATHY →					
JSPE-S	0.32	0.08	4.20	<.001	0.17 - 0.47
QCAE - Cognitive	0.97	0.11	8.72	<.001	0.75 - 1.18
QCAE - Affective	0.38	0.06	6.73	<.001	0.27 - 0.49
AMSP	0.39	0.05	8.33	<.001	0.30 - 0.48
**MODEL 2 – ABILITY TO RECOGNIZE OTHERS' EMOTIONS (N=466)**					
**Structural**					
OSCE ←					
GERT-S	0.15	0.05	3.13	.002	0.06 - 0.25
**Measurement**					
OSCE →					
Final total points	0.84	0.02	36.65	<.001	0.79 - 0.88
Communication competency	0.82	0.02	39.11	<.001	0.78 - 0.86
Communication skills grid	0.75	0.03	28.56	<.001	0.70 - 0.80
**MODEL 3 – SIMULATED PATIENT (N=435)**					
**Structural**					
OSCE ←					
SIMULATED PATIENT	0.54	0.11	5.07	.000	0.33 - 0.76
**Measurement**					
OSCE →					
Final total points	0.83	0.02	36.15	<.001	0.79 - 0.88
Communication competency	0.82	0.02	38.93	<.001	0.78 - 0.86
Communication skills grid	0.77	0.03	29.02	<.001	0.72 - 0.82
SIMULATED PATIENT →					
SP3.1	0.38	0.07	5.56	<.001	0.24 - 0.51
SP3.2	0.55	0.10	5.48	<.001	0.35 - 0.75
SP3.1 →					
JSPPPE	0.92	0.01	68.69	<.001	0.89 - 0.94
Satisfaction with communication	0.92	0.01	85.84	<.001	0.90 - 0.95
Communication skills grid	0.87	0.01	58.33	<.001	0.84 - 0.90
SP3.2 →					
JSPPPE	0.85	0.02	46.36	<.001	0.82 - 0.89
Satisfaction with communication	0.92	0.01	72.71	<.001	0.90 - 0.95
Communication skills grid	0.89	0.02	57.76	<.001	0.86 - 0.93

Goodness of fit indices for Model 1: CFI = .968, RMSEA = .066, SRMR = .049, Chi^2 = 39.073***; Model 2: CFI = .997, RMSEA = .041, SRMR = .015, Chi^2 = n.s.; Model 3: CFI = .989, RMSEA = .052, SRMR = .029, Chi^2 = 52.05**. Abbreviations: SP = simulated patient evaluations; OSCE = Objective Structured Clinical Examination; JSPE-S = Jefferson Scale of Physician Empathy - Student version; QCAE = Questionnaire of Cognitive and Affective Empathy; AMSP = Ability to Modify Self-Presentation; GERT-S = Geneva Emotion Recognition Test - Short Version; JSPPPE = Jefferson Scale of Patient Perception of Physician Empathy; CFI = Comparative Fit Index; RMSEA = Root Mean Square Error of Approximation; SRMR = Standardized Root Mean Square Residual.

Supplementary Table 4. Detailed results of Year 5 cohort's structural equation models with self-reported empathy, ability to recognize others' emotions, and simulated patient evaluations predicting independently exam performance.

	Std. Coef.	SE	z	p	95% CI		
**MODEL 1 – SELF-REPORTED EMPATHY (N=398)**							
**Structural**							
OSCE ←							
SELF-REPORTED EMPATHY	0.15	0.07	2.06	.039	0.01	-	0.30
**Measurement**							
OSCE →							
Final total points	0.77	0.02	31.70	<.001	0.73	-	0.82
Communication competency	0.95	0.02	44.03	<.001	0.91	-	0.99
Communication skills grid	0.73	0.03	24.55	<.001	0.67	-	0.79
SELF-REPORTED EMPATHY →							
JSPE-S	0.37	0.08	4.64	<.001	0.22	-	0.53
QCAE - Cognitive	0.86	0.09	9.45	<.001	0.68	-	1.04
QCAE - Affective	0.33	0.06	5.28	<.001	0.21	-	0.46
AMSP	0.47	0.06	8.56	<.001	0.36	-	0.58
**MODEL 2 – ABILITY TO RECOGNIZE OTHERS' EMOTIONS (N=398)**							
**Structural**							
OSCE ←							
GERT-S	0.13	0.05	2.48	.013	0.03	-	0.24
**Measurement**							
OSCE →							
Final total points	0.78	0.02	32.36	<.001	0.73	-	0.83
Communication competency	0.94	0.02	44.54	<.001	0.90	-	0.98
Communication skills grid	0.74	0.03	25.40	<.001	0.68	-	0.79
**MODEL 3 – SIMULATED PATIENT (N=393)**							
**Structural**							
OSCE ←							
SIMULATED PATIENT	0.21	0.05	3.85	<.001	0.10	-	0.32
**Measurement**							
OSCE →							
Final total points	0.78	0.02	31.87	<.001	0.73	-	0.83
Communication competency	0.95	0.02	47.22	<.001	0.91	-	0.99
Communication skills grid	0.73	0.03	25.23	<.001	0.68	-	0.79
SIMULATED PATIENT →							
JSPPPE	0.91	0.01	74.44	<.001	0.89	-	0.94
Satisfaction with communication	0.93	0.01	88.22	<.001	0.91	-	0.95
Communication skills grid	0.92	0.01	72.61	<.001	0.89	-	0.94

Goodness of fit indices for Model 1: CFI = .960, RMSEA = .077, SRMR = .056, Chi^2 = 43.52***; Model 2: CFI = .991, RMSEA = .079, SRMR = .024, Chi^2 = 6.98*; Model 3: CFI = .997, RMSEA = .040, SRMR = .024, Chi^2 = n.s.
Abbreviations: SP = simulated patient evaluations; OSCE = Objective Structured Clinical Examination; JSPE-S = Jefferson Scale of Physician Empathy - Student version; QCAE = Questionnaire of Cognitive and Affective Empathy; AMSP = Ability to Modify Self-Presentation; GERT-S = Geneva Emotion Recognition Test - Short Version; JSPPPE = Jefferson Scale of Patient Perception of Physician Empathy; CFI = Comparative Fit Index; RMSEA = Root Mean Square Error of Approximation; SRMR = Standardized Root Mean Square Residual.

Supplementary Table 5. Detailed results of Year 3 cohort's structural equation models with self-reported empathy, ability to recognize others' emotions, and simulated patient evaluations predicting conjointly exam performance (N=432).

	Std. Coef.	SE	z	p	95% CI		
**Structural**							
OSCE ←							
SELF-REPORTED EMPATHY	0.09	0.06	1.54	.124	-0.02	-	0.20
GERT-S	0.02	0.05	0.41	.684	-0.07	-	0.11
SIMULATED PATIENT	0.49	0.12	4.09	.000	0.25	-	0.72
Identifying as male	-0.14	0.07	-1.92	.055	-0.28	-	0.00
Age	-0.11	0.06	-1.74	.082	-0.24	-	0.01
parents' education	0.13	0.05	2.51	.012	0.03	-	0.23
social support	0.14	0.06	2.27	.023	0.02	-	0.25
having a partner	0.07	0.06	1.18	.238	-0.05	-	0.19
physical activity	0.03	0.06	0.43	.669	-0.09	-	0.15
having consulted a psy	-0.01	0.06	-0.18	.855	-0.13	-	0.11
SELF-REPORTED EMPATHY ←							
Identifying as male	-0.20	0.09	-2.32	.020	-0.37	-	-0.03
Age	0.13	0.05	2.86	.004	0.04	-	0.23
parents' education	0.00	0.06	-0.08	.935	-0.12	-	0.11
social support	0.19	0.06	3.22	.001	0.08	-	0.31
having a partner	0.11	0.06	1.76	.078	-0.01	-	0.23
physical activity	0.06	0.06	1.00	.318	-0.05	-	0.16
having consulted a psy	0.08	0.08	1.02	.306	-0.07	-	0.23
GERT-S ←							
Identifying as male	-0.20	0.05	-4.22	<.001	-0.29	-	-0.11
Age	0.01	0.05	0.23	.817	-0.08	-	0.10
parents' education	0.07	0.05	1.51	.131	-0.02	-	0.16
social support	0.15	0.05	3.09	.002	0.05	-	0.24
having a partner	0.04	0.05	0.93	.355	-0.05	-	0.14
physical activity	0.01	0.05	0.17	.862	-0.09	-	0.10
having consulted a psy	-0.02	0.05	-0.33	.738	-0.11	-	0.08
SIMULATED PATIENT ←							
Identifying as male	-0.26	0.09	-2.93	.003	-0.44	-	-0.09
Age	0.16	0.09	1.81	.071	-0.01	-	0.33
parents' education	-0.01	0.08	-0.08	.933	-0.16	-	0.15
social support	0.05	0.08	0.62	.535	-0.11	-	0.21
having a partner	0.16	0.08	1.97	.049	0.00	-	0.32
physical activity	-0.03	0.09	-0.36	.715	-0.20	-	0.14
having consulted a psy	-0.02	0.09	-0.28	.782	-0.20	-	0.15

Supplementary Table 5 (continued)

	Std. Coef.	SE	z	p	95% CI		
**Measurement**							
OSCE →							
Final total points	0.82	0.02	35.67	<.001	0.78	-	0.87
Communication competency	0.83	0.02	38.33	<.001	0.79	-	0.87
Communication skills grid	0.77	0.03	28.61	<.001	0.71	-	0.82
SELF-REPORTED EMPATHY →							
JSPE-S	0.44	0.08	5.68	<.001	0.29	-	0.60
QCAE - Cognitive	0.80	0.11	7.55	<.001	0.60	-	1.01
QCAE - Affective	0.49	0.09	5.71	<.001	0.32	-	0.66
AMSP	0.37	0.08	4.89	<.001	0.22	-	0.52
SIMULATED PATIENT →							
SP3.1	0.39	0.07	5.45	<.001	0.25	-	0.53
SP3.2	0.53	0.10	5.21	<.001	0.33	-	0.73
SP3.1 →							
JSPPPE	0.92	0.01	68.43	<.001	0.89	-	0.94
Satisfaction with communication	0.93	0.01	85.51	<.001	0.90	-	0.95
Communication skills grid	0.87	0.02	57.70	<.001	0.84	-	0.90
SP3.2 →							
JSPPPE	0.85	0.02	45.81	<.001	0.81	-	0.89
Satisfaction with communication	0.92	0.01	71.36	<.001	0.90	-	0.95
Communication skills grid	0.89	0.02	56.86	<.001	0.86	-	0.93

Goodness of fit indices: CFI = .958; RMSEA = .046; SRMR = .046; Chi^2 = 271.94***. Abbreviations: SP = simulated patients' evaluations; OSCE = Objective Structured Clinical Examination; JSPE-S = Jefferson Scale of Physician Empathy - Student version; QCAE = Questionnaire of Cognitive and Affective Empathy; AMSP = Ability to Modify Self-Presentation; GERT-S = Geneva Emotion Recognition Test - Short Version; JSPPPE = Jefferson Scale of Patient Perception of Physician Empathy.

Supplementary Table 6. Detailed results of Year 5 cohort's structural equation models with self-reported empathy, ability to recognize others' emotions, and simulated patient evaluations predicting conjointly exam performance (N=392).

	Std. Coef.	SE	z	p	95% CI		
**Structural**							
OSCE ←							
SELF-REPORTED EMPATHY	0.11	0.07	1.55	.121	-0.03	-	0.25
GERT-S	0.05	0.05	1.06	.291	-0.05	-	0.16
SIMULATED PATIENT	0.20	0.05	3.76	<.001	0.10	-	0.31
Identifying as male	-0.19	0.06	-3.41	.001	-0.30	-	-0.08
Age	0.04	0.05	0.89	.374	-0.05	-	0.14
parents' education	0.08	0.05	1.55	.122	-0.02	-	0.18
social support	0.00	0.05	0.07	.945	-0.09	-	0.10
having a partner	0.07	0.05	1.28	.202	-0.04	-	0.17
physical activity	0.01	0.05	0.27	.791	-0.09	-	0.12
having consulted a psy	-0.01	0.05	-0.17	.866	-0.11	-	0.09
SELF-REPORTED EMPATHY ←							
Identifying as male	-0.11	0.09	-1.24	.215	-0.29	-	0.07
Age	0.10	0.06	1.59	.112	-0.02	-	0.22
parents' education	0.05	0.06	0.79	.431	-0.07	-	0.17
social support	0.13	0.07	1.97	.049	0.00	-	0.26
having a partner	0.06	0.07	0.85	.396	-0.08	-	0.19
physical activity	0.13	0.07	1.78	.075	-0.01	-	0.26
having consulted a psy	0.14	0.06	2.15	.031	0.01	-	0.26
GERT-S ←							
Identifying as male	-0.17	0.05	-3.11	.002	-0.27	-	-0.06
Age	-0.05	0.05	-1.12	.261	-0.14	-	0.04
parents' education	0.01	0.05	0.21	.832	-0.09	-	0.11
social support	0.00	0.06	0.08	.939	-0.10	-	0.11
having a partner	0.08	0.06	1.48	.138	-0.03	-	0.19
physical activity	-0.04	0.06	-0.72	.469	-0.15	-	0.07
having consulted a psy	0.08	0.05	1.58	.113	-0.02	-	0.18
SIMULATED PATIENT ←							
Identifying as male	-0.07	0.05	-1.23	.220	-0.17	-	0.04
Age	-0.07	0.04	-1.81	.070	-0.15	-	0.01
parents' education	-0.11	0.05	-2.12	.034	-0.21	-	-0.01
social support	0.04	0.05	0.87	.384	-0.06	-	0.14
having a partner	0.07	0.05	1.41	.158	-0.03	-	0.18
physical activity	0.08	0.05	1.63	.103	-0.02	-	0.17
having consulted a psy	0.09	0.05	1.90	.057	0.00	-	0.19

Supplementary Table 6 (continued)

	Std. Coef.	SE	z	p	95% CI		
**Measurement**							
OSCE →							
Final total points	0.78	0.02	32.91	<.001	0.74	-	0.83
Communication competency	0.94	0.02	46.97	<.001	0.90	-	0.98
Communication skills grid	0.74	0.03	25.62	<.001	0.68	-	0.79
SELF-REPORTED EMPATHY →							
JSPE-S	0.39	0.09	4.32	<.001	0.21	-	0.57
QCAE - Cognitive	0.81	0.11	7.59	<.001	0.60	-	1.02
QCAE - Affective	0.35	0.09	4.02	<.001	0.18	-	0.52
AMSP	0.47	0.06	8.18	<.001	0.35	-	0.58
SIMULATED PATIENT →							
JSPPPE	0.91	0.01	73.66	<.001	0.89	-	0.94
Satisfaction with communication	0.93	0.01	87.04	<.001	0.91	-	0.95
Communication skills grid	0.92	0.01	71.72	<.001	0.89	-	0.94

Goodness of fit indices: CFI = .933; RMSEA = .061; SRMR = .053; Chi^2 = 223.64***. Abbreviations: SP = simulated patients' evaluations; OSCE = Objective Structured Clinical Examination; JSPE-S = Jefferson Scale of Physician Empathy - Student version; QCAE = Questionnaire of Cognitive and Affective Empathy; AMSP = Ability to Modify Self-Presentation; GERT-S = Geneva Emotion Recognition Test - Short Version; JSPPPE = Jefferson Scale of Patient Perception of Physician Empathy.
